# The measurement performance of the EQ-5D-5L versus EQ-5D-3L in patients with hidradenitis suppurativa

**DOI:** 10.1007/s11136-020-02732-x

**Published:** 2021-02-03

**Authors:** Alex Bató, Valentin Brodszky, L. Hunor Gergely, Krisztián Gáspár, Norbert Wikonkál, Ágnes Kinyó, Ákos Szabó, Zsuzsanna Beretzky, Andrea Szegedi, Éva Remenyik, Norbert Kiss, Miklós Sárdy, Fanni Rencz

**Affiliations:** 1 Department of Health Economics, Corvinus University of Budapest, Fővám tér 8, H-1093 Budapest, Hungary; 2 Károly Rácz Doctoral School of Clinical Medicine, Semmelweis University, Üllői út 26, H-1085 Budapest, Hungary; 3 Department of Dermatology, Venereology and Dermatooncology, Faculty of Medicine, Semmelweis University, Mária u. 41, H-1085 Budapest, Hungary; 4 Faculty of Medicine, Departments of Dermatology, University of Debrecen, Nagyerdei krt. 98, H-4032 Debrecen, Hungary; 5 Faculty of Medicine, Department of Dermatological Allergology, University of Debrecen, Nagyerdei krt. 98, H-4032 Debrecen, Hungary; 6 University of Pécs Medical School Department of Dermatology, Venereology and Oncodermatology, Akác utca 1, H-7632 Pécs, Hungary; 7 Premium Postdoctoral Research Programme, Hungarian Academy of Sciences, Nádor u. 7, H-1051 Budapest, Hungary

**Keywords:** Health-related quality of life, EQ-5D-3L, EQ-5D-5L, Validity, Psychometrics, Hidradenitis suppurativa

## Abstract

**Purpose:**

Hidradenitis suppurativa (HS) is a chronic inflammatory skin disease that affects up to 1% of the population in Europe. The EQ-5D is the most commonly used generic instrument for measuring health-related quality of life among HS patients. This study aims to compare the measurement properties of the two adult versions of EQ-5D (EQ-5D-3L and EQ-5D-5L) in patients with HS.

**Methods:**

We recruited 200 consecutive patients with HS (mean age 37 years, 38% severe or very severe HS) to participate in a multicentre cross-sectional survey. Patients completed the EQ-5D-3L, EQ-5D-5L, Dermatology Life Quality Index (DLQI) and Skindex-16 questionnaires.

**Results:**

More than twice as many different health state profiles occurred in the EQ-5D-5L compared to the EQ-5D-3L (101 vs. 43). A significant reduction in ceiling effect was found for the mobility, self-care and usual activities dimensions. A good agreement was established between the EQ-5D-3L and EQ-5D-5L with an intraclass correlation coefficient of 0.872 (95% CI 0.830–0.903; *p* < 0.001) that was confirmed by a Bland-Altman plot. EQ-5D-5L improved both the absolute and relative informativity in all dimensions except for anxiety/depression. EQ-5D-3L and EQ-5D-5L demonstrated similar convergent validity with DLQI and Skindex-16. EQ-5D-5L was able to better discriminate between known groups of patients based on the number of comorbidities and disease severity (HS-Physician's Global Assessment).

**Conclusion:**

In patients with HS, the EQ-5D-5L outperformed the EQ-5D-3L in feasibility, ceiling effects, informativity and known-groups validity for many important clinical characteristics. We recommend using the EQ-5D-5L in HS patients across various settings, including clinical care, research and economic evaluations.

## Introduction

Hidradenitis suppurativa (HS) is a chronic, inflammatory, recurrent skin disease that usually starts after puberty with painful, deep-seated lesions [1]. It typically affects the apocrine gland-bearing areas of the body, most commonly the axillary, inguinal and anogenital regions [2]. An average prevalence of up to 1% and a mean incidence of 6.0 per 100,000 person‐years have been reported in Europe [3–6]. Observed comorbidities fall into several categories: cardiovascular diseases, inflammatory and autoimmune diseases, hormone-related disorders and psychiatric illnesses [7, 8]. Therapeutic approaches currently include the use of topical therapies, systemic antibiotics, hormonal therapies, surgical options and biologics, such as adalimumab and infliximab (the latter currently off-label) [2, 9]. Patients often experience a substantial diagnostic delay of up to 7 years that represents a serious burden to both patients and healthcare systems [10–13].

Patients are likely to have a severely impaired health-related quality of life (HRQoL) due to the clinical symptoms of HS, associated comorbidities and side effects of treatments [14–16]. While disease-specific (e.g. HS Quality of Life [HS-QoL and HiSQOL] and HIDRAdisk [17–20]) and skin-specific HRQoL measures (e.g. Dermatology Life Quality Index, DLQI and Skindex instruments) are widely used in patients with HS [15], less empirical research has been conducted on general HRQoL in this patient population. The most commonly used instrument for measuring general HRQoL among HS patients is the EQ-5D questionnaire [15]. A main advantage of the EQ-5D health status measure is that it can be used to derive preference-based index scores for economic evaluations of health interventions [21]. In many countries, including the US, Canada, and numerous European countries, the EQ-5D is the recommended outcome measure to estimate quality-adjusted life years (QALYs) in cost-effectiveness analyses [22, 23]. Furthermore, being a generic HRQoL measure, the EQ-5D allows comparisons with general population normative data and across various patient populations, also outside of dermatology.

The economic burden of HS has received an increasing attention from healthcare providers and policymakers since 2015, when a biological drug, adalimumab was introduced in the treatment of HS [24–26]. In the US, for example, seven-month costs associated with adalimumab therapy amount to $63,953 (in 2018 prices) [27, 28]. Cost-effectiveness analyses weigh the costs and benefits (i.e. HRQoL improvement) of new treatments to guide financial decisions in healthcare. Having a central role in these analyses, EQ-5D results can help to demonstrate the value of these treatments, and hence, to improve patients’ access to new treatment approaches.

There are two adult versions of the EQ-5D, the EQ-5D-3L (hereafter 3L) developed in 1990 and the newer, EQ-5D-5L (hereafter 5L) that has been available since 2009 [29, 30]. A number of studies applied and validated the 3L in HS patients, whereas the use of the 5L is less common in this patient population [31–36]. So far, no head-to-head comparison studies have been performed to compare the measurement properties of the 3L and 5L among patients with skin diseases other than psoriasis [37–39].

The objective of the current study was to compare the measurement properties of the 3L and 5L descriptive systems of the EQ-5D in a common sample of patients with HS. We aim to test the following measurement properties: feasibility, agreement, ceiling effects, redistribution properties, inconsistency in responses, informativity, convergent and known-groups validity.

## Methods

### Study design and patient population

Between September 2017 and October 2019, we carried out a cross-sectional survey at three academic dermatology clinics in Hungary [40]. The inclusion criteria to this study were as follows: (i) ≥ 18 years of age; (ii) cognitive ability to understand the questionnaire; (iii) diagnosis of HS by a dermatologist; and (iv) signing a consent form prior to the data collection. The Scientific and Ethical Committee of the Medical Research Council in Hungary granted permission for conducting this study (ref. #40579-2/2017/EKU).

Data were collected through paper-based questionnaires completed by patients and their dermatologists. Patients were asked about socio-demographic characteristics, general health status and HRQoL. Each patient rated their current and worst HS-related pain intensity on a 10-cm-long, horizontal VAS from 0 (no pain at all) to 10 (pain as bad as it could be). Patients were also asked to assess their severity using the Patient’s Global Assessment (PtGA) VAS providing a range of scores from 0 to 100, where 0 indicated ‘not severe at all’ and 100 represented ‘very severe’.

Dermatologists provided information about clinical characteristics, comorbidities and affected body sites (i.e. localisation). Disease severity was evaluated by the following three measures: Hurley staging [41], HS-Physician’s Global Assessment (HS-PGA) [42] and Modified Sartorius Score [43].

### Health-related quality of life measures

We used the validated Hungarian versions of standardised HRQoL measures. We measured general HRQoL by using the 3L, 5L and EQ visual analogue scale (EQ VAS). Following prior work [44], patients filled in the 5L before the 3L, in order to prevent the underuse of levels 2 and 4 on the 5L, and two skin-specific questionnaires were placed between the 5L and the 3L. The EQ VAS was completed only once (that of the 5L version).

For quantifying skin-specific HRQoL, we used Dermatology Life Quality Index (DLQI) and Skindex-16. These two skin-specific instruments assess quite different areas of HRQoL: while the DLQI mainly focuses on functional impairments, Skindex-16 is considered better at capturing the emotional and mental aspects of the skin disease [45, 46].

### EQ-5D-3L and EQ-5D-5L

The EQ-5D is a generic measure of health status that comprises of two parts: a descriptive system and the EQ VAS [29]. The descriptive system focuses on five dimensions of health (mobility, self-care, usual activities, pain/discomfort and anxiety/depression). The EQ VAS records self-rated health on a 20-cm-long vertical health thermometer anchored at 0 (‘the worst health you can imagine’) to 100 (‘the best health you can imagine’). The timeframe of the questionnaire is the day of the completion (i.e. ‘your health today’.)

The 3L has three response levels for each dimension (no problems = 1, some/moderate problems = 2, extreme problems/unable to/confined to bed = 3) providing 243 unique health states [29]. The 5L has five levels for each dimension (no problems = 1, slight problems = 2, moderate problems = 3, severe problems = 4 and unable to/extreme problems = 5) allowing a total of 3125 distinct health states [30]. Note that wording of the most severe level of mobility in 3L ‘confined to bed’ is changed to ‘unable to walk about’ in the 5L, and the middle levels are also standardised to consistently use ‘moderate’ in all dimensions of the 5L. In addition to these changes, there are a number of other minor differences between the Hungarian 3L and 5L versions that affect both modifiers [e.g. ‘very strong’(3L) vs. ‘extreme’(5L)] and descriptors [e.g. ‘anxiety/feeling down’(3L) vs. ‘anxiety/depression’(5L)] [47].

We computed index scores using the Hungarian value sets, where 3L and 5L index scores have been derived parallel from a representative sample of the Hungarian general population using composite time trade-off approach [47]. The scoring range for the 3L and 5L are from 1 (‘11111’, full health) to − 0.865 (‘33333’ on the 3L) and − 0.848 (‘55555’ on the 5L), respectively. An index score of zero indicates dead, and negative values represent health states worse than dead.

#### Dermatology Life Quality Index (DLQI)

DLQI is the most widely used HRQoL instrument in clinical practice and research in patients with skin diseases [48]. It consists of 10 items covering the following aspects of health over the past week: symptoms and feelings, daily activities, leisure, work or school, personal relationships and treatment. Each item is scored on a four-point scale: ‘not at all’ or ‘not relevant’ = 0, ‘a little’ = 1, ‘a lot’ = 2 and ‘very much’ = 3. DLQI total score is calculated by summing the score of each item, resulting in a maximum score of 30, where a higher score refers to a worse HRQoL.

#### Skindex-16

Skindex-16 is skin-specific HRQoL instrument with a one-week recall period [49]. It comprises 16 items, each is rated on a 7-item bipolar scale, where the endpoints are labelled ‘never bothered’ and ‘always bothered’. The responses are categorised into three subscales: symptoms (items 1–4), emotions (items 5–11) and functioning (items 12–16). Subscale scores are transformed to a linear scale of 0-100, where higher scores indicate more impaired levels of HRQoL.

### Statistical analyses

Data analysis was built on previous studies that compared the measurement properties of the 3L and 5L in other patient and general population samples [39, 44, 50–52].

#### Feasibility and ceiling effects

Feasibility was determined by examining the percentage of missing responses by dimension. The proportion of missing data was documented, and no missing imputation technique was performed. Histograms were plotted to visualise the empirical distributions of the 3L and 5L index scores. To assess ceiling effects, we computed the proportion of patients having ‘no problems’ on one or all dimensions (‘11111’) and compared them between the 3L and 5L by using McNemar’s test. We expected a reduction in those selecting ‘no problems’ for the 5L compared to the 3L due to the two extra response levels. Both absolute and relative (%) reduction in ceiling effects were determined when moving from 3L to 5L.

#### Agreement

A Bland-Altman plot was drawn to visualise the agreement between the 3L and 5L [53]. The intraclass correlation coefficient (ICC) was used as an index of parallel forms reliability that reflects both the degree of correlation and agreement between the 3L and 5L [54]. The ICC represents the proportion of total variance that is attributable to the differences between individuals, as opposed to the differences between the two instruments (3L and 5L). ICC values were calculated using a two-way random model with absolute agreement [55]. We rated ICC values as poor if 0–0.39, fair if 0.40–0.59, good if 0.60–0.74 and excellent if 0.75–1 [56].

#### Redistribution properties

We assessed redistribution properties from 3L to 5L by cross-tabulating 3L-5L response pairs. We computed the proportion of consistent and inconsistent 3L-5L response pairs. We considered levels 1, 3 and 5 in the 5L descriptive system matched pairs of the 1, 2 and 3 levels in the 3L. Thus, 3L responses differing at least two levels from their 5L pairs were considered ‘inconsistent’ [44]. For example, a patient reports ‘some problems washing or dressing’ (level 2) in the 3L and ‘unable to wash or dress’ in the 5L (level 5). The average size of inconsistency was assessed according to the following weights: 0 = 3L responses are not more than one level away from their 5L pairs, 1 = 3L responses are two levels away from their 5L pairs, 2 = 3L responses are three levels away from their 5L pairs and 3 = 3L responses are four levels away from their 5L pairs [44].

#### Informativity

We hypothesised that the 5L with its two extra levels improves discriminatory power of the 3L in terms of informativity [57]. Shannon’s (H') and Shannon’s evenness (J') indices were used to assess informativity of each of the five dimensions [58, 59]. H’ represents the extent to which the information is evenly distributed across all responses. J' combines the evenness of a distribution and the number of response levels used. H' and J' were calculated using the following formulas, where L refers to the number of levels in a dimension of the descriptive system, and p_i_ is the proportion of patients reporting their health in the *i*th level:

$${H}^{^{\prime}}=-\sum_{i=1}^{L}{p}_{i}{log}_{2}{p}_{i}$$

$$J^{\prime} = \frac{{H^{\prime}}}{{H^{\prime}_{{\max }} }},\;{\text{where}}\,\,{\mkern 1mu} H^{\prime}_{{{\text{max}}}} {\text{ = log}}_{2} {\text{L}}.$$

H′ values range between 0 and log_2_L (= 1.58 for the 3L and 2.32 for the 5L), where higher the H′, the more informative the item and the better the discriminatory power are. J′ can take values between 0 (i.e. all responses are concentrated in one response level; worst discriminatory power) and 1 (i.e. even distribution across response levels; best discriminatory power) [39].

#### Convergent and validity

Spearman’s rank order correlations were conducted to explore the convergent validity of the five dimensions and index scores with other scales. We hypothesised the EQ-5D dimensions and index scores to moderately correlate with EQ VAS, DLQI and Skindex-16, and weakly with disease severity measures, including HS-PGA, Modified Sartorius Score and PtGA VAS [31, 33, 35]. For pain scales, we hypothesised a moderate or strong correlation with the pain/discomfort dimension and index scores of the EQ-5D and a weak correlation with all other dimensions [60–62]. Correlation coefficients (*ρ*) were interpreted as very weak (< 0.20), weak (0.20–0.39), moderate (0.40–0.59) and strong correlation (0.60 ≤) [63].

#### Known-groups validity

Known-groups validity was examined by comparing subsets of patients defined based on clinical characteristics. We hypothesised that patients with higher body mass index (BMI), with more comorbidities, inguinal or perianal localisation or higher disease severity have lower EQ-5D index scores [31–33, 35, 64–67]. We used Mann–Whitney and Kruskal Wallis tests to look for differences in 3L and 5L index scores between groups. For each known group, we estimated effect sizes according to the following formulas:

$$ES\left( Z \right) = \frac{{{\text{Mann - Whitney}} Z}}{n - 1}\;{\text{and}}$$

$$ES\left( H \right) = \frac{{{\text{Kruskal}} - {\text{Wallis}}\,{ }H - k + 1}}{n - k},$$

where k denotes the number of groups and n refers to the sample size. Effect size values were considered as small if ≥ 0.01, moderate if ≥ 0.06 and large if ≥ 0.14 [68]. Then, relative efficiency (RE) was computed as the ratio of the ESs of the 5L and 3L index scores. A RE value of > 1 indicated that the 5L was more efficient in discriminating between known groups compared to the 3L. All the statistics were two-sided, and *p* < 0.05 was considered statistically significant. Data were analysed with Stata 13 (StataCorp, TX, USA).

## Results

### Patient characteristics

A total of 200 consecutive patients with HS participated in the survey (Table 1). The majority of the patients were male (61.5%), and the mean age was 37.13 ± 12.43 years. Mean disease duration was 4.76 ± 6.72 years. Overall, 46.0% of the patients had at least one comorbidity, with cardiovascular disease (16.5%), acne vulgaris (7.0%), inflammatory bowel disease (7.0%), diabetes (6.0%) and psychiatric illness (6.0%) being the most commonly reported. A total of 80.7% of the patients were overweight or obese (BMI ≥ 25). Almost half of the patients had Hurley III stage disease (48.5%). According to HS-PGA scores, over one-third of the patients had severe or very severe HS (Table 1).

**Table 1:** Characteristics of patients with HS

Variables	Mean (SD) or N (%)
Age (years)	37.13 (12.43)
Sex	
Female	77 (38.5%)
Male	123 (61.5%)
Disease duration (years)	4.76 (6.72)
Disease severity	
Hurley staging (*missing n* = *4*)	
Hurley I	22 (11.2%)
Hurley II	79 (40.3%)
Hurley III	95 (48.5%)
HS-PGA (*missing n* = *7*)	
Clear	6 (3.1%)
Minimal	7 (3.6%)
Mild	37 (19.3%)
Moderate	69 (35.9%)
Severe	40 (20.7%)
Very severe	34 (17.7%)
Modified Sartorius Score^a^ (*missing n* = *2*)	60.69 (50.24)
PtGA VAS (0-100) (*missing n* = *1*)	69.62 (22.22)
Current pain intensity VAS (0–10) (*missing n= 1*)	4.70 (2.99)
Worst pain intensity^*b*^ VAS (0–10) (*missing n= 1*)	6.28 (3.04)
Health-related quality of life	
EQ-5D-3L index (− 0.865 to 1) (*missing n* = *2*)	0.78 (0.21)
EQ-5D-5L index (− 0.848 to 1) (*missing n* = *2*)	0.76 (0.30)
EQ VAS (0–100) (*missing n* = *2*)	64.29 (22.68)
DLQI (0–30) (*missing n* = *2*)	11.75 (8.11)
Skindex-16 (*missing n* = *2*)	
Symptoms subscale (0–100)	46.74 (29.36)
Emotions subscale (0–100)	64.55 (29.28)
Functioning subscale (0–100)	49.40 (34.70)

For EQ-5D-5L and EQ VAS higher scores refer to better health status. for all other measures higher scores represent worse health status

*DLQI* Dermatology Life Quality Index, *HS* hidradenitis suppurativa, *HS-PGA* Physicians’ Global Assessment of HS severity, *PtGA VAS* Patient's Global Assessment of disease severity visual analogue scale

^a^The measure has no upper limit

^b^For the past one month

### Feasibility

**Fig. 1:**
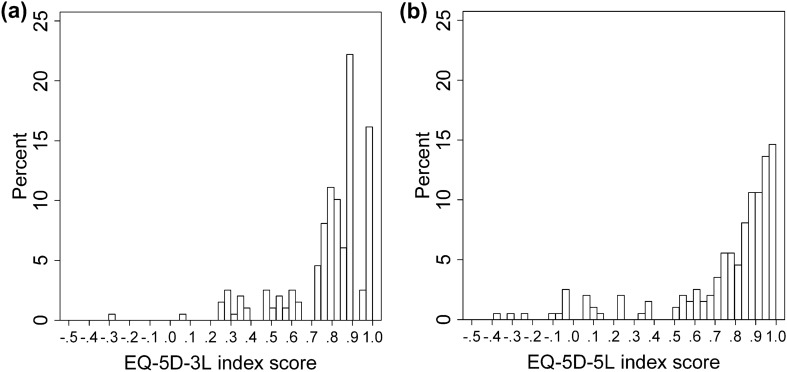
Distribution of EQ-5D-3L and EQ-5D-5L index scores

One patient did not complete the 5L questionnaire, and there were two partially incomplete 3L and one 5L descriptive systems. There were two missing values on the EQ VAS. For 3L, 43 distinct health state profiles were observed versus 101 for the 5L. There was a great dispersion of both 3L and 5L profiles among HS patients with few clustering. One and 10 patients had negative index scores in the 3L and 5L, respectively. There were more patients between index scores of 0.2 to 0.6 and 0.7 to 0.8 with the 3L, whereas the 5L allowed more observations for mild (index score 0.9-1) and very severe health states (index score < 0.2) (Fig.1).

### Ceiling effects

Patients reported the most problems with pain/discomfort (‘any problems’: 75.4% in 3L and 77.4% in 5L), while the least problems occurred with self-care (‘no problems’: 19.5% in 3L and 18.3% in 5L) (Table 2). Absolute reduction in ceiling effects was the highest for self-care (8.8%), whereas relative reduction was the highest for usual activities (15.5%). We found increased ceiling effects for the 5L in the dimension of anxiety/depression with absolute and relative increases of 5.0% and 11.4%, respectively. Ceiling effect reduction was statistically significant for the mobility, self-care and usual activities dimensions. The proportion of ‘11111’ profiles decreased from 16.0% on the 3L to 14.6% on the 5L. The absolute and relative ceiling effect reductions in the proportion of full health (‘11111’) responses were 1.4% and 9.4%, respectively. There were four (2.0%) ‘the best health you can imagine’ (= 100) and no ‘the worst health you can imagine’ (= 0) responses on the EQ VAS.

**Table 2:** Ceiling effects, inconsistencies and informativity

Dimensions	Ceiling effects							Inconsistencies		Informativity			
	EQ-5D-3L		EQ-5D-5L		Ceiling effect reduction		McNemar’s test *p* value			EQ-5D-3L		EQ-5D-5L	
	*n*	Ceiling, *n*, %	*n*	Ceiling, *n*, %	Absolute	Relative (%)		Inconsistent response pairs (*n*, %)^*a*^	Average size of inconsistencies	H'	J'	H'	J'
Mobility	199	121 (60.8%)	199	107 (53.8%)	7.0%	11.6%	0.003	7 (3.5%)	1.14	0.93	0.59	1.65	0.71
Self-care	200	161 (80.5%)	198	142 (71.7%)	8.8%	11.8%	< 0.001	7 (3.5%)	1.14	0.71	0.45	1.25	0.54
Usual activities	200	103 (51.5%)	198	87 (43.9%)	7.6%	15.5%	0.024	20 (10.0%)	1.00	1.22	0.77	1.88	0.81
Pain/discomfort	199	49 (24.6%)	199	45 (22.6%)	2.0%	8.2%	0.503	15 (7.5%)	1.00	1.32	0.83	2.06	0.89
Anxiety/depression	199	88 (44.2%)	199	98 (49.2%)	-5.0%	-11.4%	0.163	30 (15.1%)	1.20	1.38	0.87	1.79	0.77
Overall (11111) or mean	200	32 (16.0%)	199	29 (14.6%)	1.40%	9.38%	0.607	79 (8.0%)	1.10	1.11	0.70	1.73	0.74

*H'* Shannon’s index, *J'* Shannon’s evenness index

^**a**^The total number of pairs is 198 for all dimensions

### Agreement

**Fig. 2:**
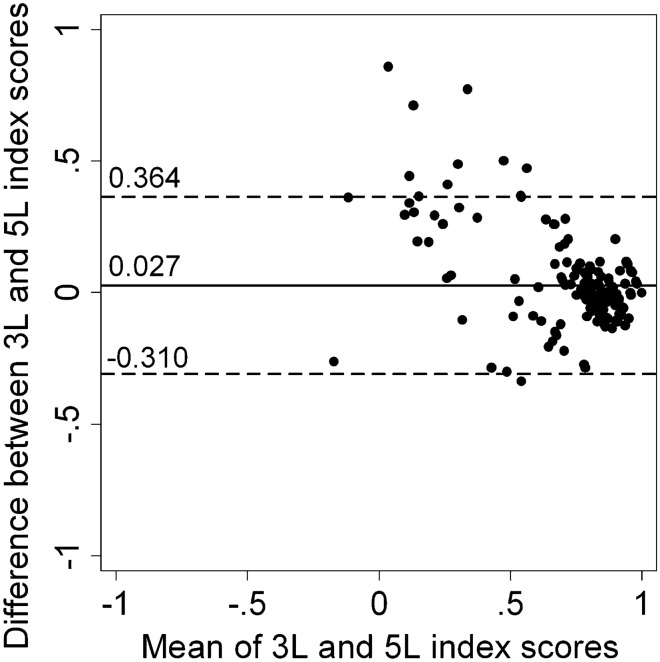
Bland-Altman plot of the EQ-5D-3L and EQ-5D-5L index scores in HS. The horizontal line represents the mean of the differences (d) between 3L and 5L index scores, while the 95% limits of agreement, obtained as d ± 1.96 *SD of d, are indicated by dashed lines

A good agreement was established between the 3L and 5L with an ICC of 0.872 (95% CI 0.830–0.903; *p* < 0.001). This finding was supported by the Bland-Altman plot (Fig. 2). Mean 3L index scores of HS patients were higher than those of the 5L (0.78 ± 0.21 and 0.76 ± 0.30; *p* < 0.031). Differences between 3L and 5L index scores tended to increase at lower mean index scores. Below the index score of 0.5, a higher 3L index score was found for almost all 3L-5L index score pairs falling out of the 95% limits of agreement.

### Redistribution properties and inconsistencies

Responses covered nearly all levels for both EQ-5D versions (Table 3). There were 79 (8.0%) inconsistent response pairs, provided by 21 (10.5%) patients. The size of inconsistency was generally low, ranging from 1.00 (self-care, usual activities and pain/discomfort) to 1.2 (anxiety/depression). The rate of inconsistent 3L-5L response pairs varied between 3.5% (mobility and self-care) and 15.1% (anxiety/depression) (Table 2).

**Table 3:** Redistribution properties: cross-tabulation of EQ-5D-3L and EQ-5D-5L responses

3L	5L				
Dimensions	Level 1	Level 2	Level 3	Level 4	Level 5
Mobility, *n* (%)					
Level 1	104 (86.0%)	13 (10.7%)	3 (2.5%)	1 (0.8%)	0 (0.0%)
Level 2	3 (3.9%)	28 (36.8%)	36 (47.4%)	9 (11.8%)	0 (0.0%)
Level 3	0 (0.0%)	0 (0.0%)	0 (0.0%)	1 (100.0%)	0 (0.0%)
Self-care, *n* (%)					
Level 1	141 (88.1%)	13 (8.1%)	5 (3.1%)	1 (0.6%)	0 (0.0%)
Level 2	1 (2.6%)	16 (42.1%)	15 (39.5%)	6 (15.8%)	0 (0.0%)
Level 3	0 (0.0%)	0 (0.0%)	0 (0.0%)	0 (0.0%)	0 (0.0%)
Usual activities, *n* (%)					
Level 1	75 (73.5%)	22 (21.6%)	5 (4.9%)	0 (0.0%)	0 (0.0%)
Level 2	12 (13.6%)	35 (39.8%)	25 (28.4%)	15 (17.0%)	1 (1.1%)
Level 3	0 (0.0%)	0 (0.0%)	2 (25.0%)	4 (50.0%)	2 (25.0%)
Pain/discomfort, *n* (%)					
Level 1	37 (75.5%)	9 (18.4%)	3 (6.1%)	0 (0.0%)	0 (0.0%)
Level 2	8 (6.6%)	58 (47.5%)	49 (40.2%)	7 (5.7%)	0 (0.0%)
Level 3	0 (0.0%)	0 (0.0%)	4 (14.8%)	16 (59.3%)	7 (25.9%)
Anxiety/depression, *n* (%)					
Level 1	76 (86.4%)	12 (13.6%)	0 (0.0%)	0 (0.0%)	0 (0.0%)
Level 2	20 (22.5%)	38 (42.7%)	25 (28.1%)	6 (6.7%)	0 (0.0%)
Level 3	1 (4.8%)	4 (19.0%)	5 (23.8%)	7 (33.3%)	4 (19.0%)

Percentages may not total 100 by row due to rounding

### Informativity

The 5L improved the absolute discriminatory power (H') of the questionnaire in all dimensions (3L: 0.71 to 1.38 vs. 5L: 1.25 to 2.06) indicating that the two extra levels of the 5L were effectively used (Table 2). Similarly, the relative discriminatory power (J') increased for all dimensions (3L: 0.45 to 0.87 vs. 5L: 0.54 to 0.89) with the exception of anxiety/depression (3L: 0.87 vs. 5L: 0.77). The average H' and J' values improved when moving from the 3L (H' = 1.11 and J' = 0.70) to the 5L (H' = 1.73 and J' = 0.74).

### Convergent validity

The results supported the majority of our hypotheses, with some interesting exceptions; for example, the EQ-5D pain/discomfort dimensions and index scores correlated strongly with the DLQI and the functioning subscale of Skindex-16 (Table 4). Furthermore, the self-care and mobility dimensions of the EQ-5D demonstrated weak correlations with the symptoms and emotions subscales of Skindex-16.

**Table 4:** Convergent validity: Spearman's correlation coefficients

Outcome measures	EQ-5D						
	Version	Mobility	Self-care	Usual activities	Pain/discomfort	Anxiety/depression	Index score
EQ VAS (0-100)	3L	− 0.406	− 0.365	− 0.350	− 0.414	− 0.449	0.535
	5L	− 0.473	− 0.399	− 0.422	− 0.505	− ***0.385***	0.592
Skindex-16 symptoms (0-100)	3L	0.331	0.287	0.420	0.523	0.422	− 0.561
	5L	0.396	0.334	***0.396***	0.595	***0.401***	− 0.573
Skindex-16 emotions (0-100)	3L	0.261	0.274	0.358	0.471	0.513	− 0.535
	5L	0.289	0.282	***0.302***	0.473	***0.511***	− ***0.500***
Skindex-16 functioning (0-100)	3L	0.403	0.434	0.538	0.610	0.566	− 0.708
	5L	0.467	0.457	***0.501***	0.625	***0.530***	− ***0.674***
DLQI (0-30)	3L	0.396	0.409	0.547	0.628	0.564	− 0.722
	5L	0.426	0.469	***0.541***	0.671	***0.560***	− ***0.697***
PtGA VAS (0-100)	3L	0.264	0.334	0.316	0.337	0.296	− 0.395
	5L	0.340	0.347	0.363	0.391	0.315	− 0.434
HS-PGA (0-5)	3L	0.291	0.348	0.371	0.230	0.205	− 0.337
	5L	0.349	***0.343***	***0.354***	0.290	***0.173***	− 0.350
Modified Sartorius Score (0-)^*a*^	3L	0.266	0.335	0.319	0.243	0.212	− 0.332
	5L	0.325	***0.301***	0.333	0.302	***0.166***	− 0.334
Current pain intensity (0–10)	3L	0.286	***0.306***	0.314	0.534	0.374	− 0.540
	5L	0.384	***0.310***	0.337	0.591	***0.315***	− 0.544
Worst average pain in the past 1 month (0–10)	3L	0.315	0.328	0.368	0.553	0.263	− 0.499
	5L	0.328	***0.299***	***0.353***	***0.529***	0.285	− ***0.473***

Bold and italic values indicate a weaker correlation for the 5L compared to the 3L

*p* < 0.05 for all correlation coefficients.

^a^The measure has no upper limit.

*DLQI* Dermatology Life Quality Index, *HS* hidradenitis suppurativa, *HS-PGA* Physicians’ Global Assessment of hs severity, *PtGA VAS* Patient's Global Assessment of disease severity visual analogue scale

When comparing the 3L and 5L, index scores of both measures showed moderate correlations with EQ VAS (0.535 vs. 0.592). The 5L exhibited stronger correlations with EQ VAS for all dimensions except for anxiety/depression (range of coefficients: − 0.449 to − 0.350 for the 3L and − 0.505 to − 0.385 for the 5L). The 5L produced stronger correlations in the dimensions of mobility, self-care and pain/discomfort with DLQI and all Skindex-16 subscale scores. However, 3L index scores correlated stronger with DLQI and all Skindex-16 subscale scores, with the exception of the symptoms subscale. Considering disease severity scales, the 5L resulted in a stronger correlation with PtGA VAS (5/5 dimensions), Modified Sartorius Score (3/5 dimensions) and HS-PGA (2/5 dimensions). The 5L demonstrated a better convergent validity with current pain intensity VAS for four dimensions, including the pain/discomfort dimension (3L: 0.534 vs. 5L: 0.591). Three dimensions of the 3L, including pain/discomfort, were better correlated with the worst pain intensity VAS scores than those of the 5L. The correlations between index scores and pain scales revealed an improved performance of the 3L and 5L with worst and current pain intensities, respectively.

### Known-groups validity

Comparisons across known groups of patients provided consistent evidence for most of our hypotheses with the exception of the impact of perianal localisation on HRQoL. Contrasting the 3L and 5L, in almost every subgroups of patients, the mean 5L index scores were lower, while the medians were higher than their respective mean and median 3L index scores (Table 5). Patients with gluteal or inguinal localisation or more severe disease, as assessed by the Hurley classification system or HS-PGA, had more impaired HRQoL on both the 3L and 5L. In addition, the 5L detected significantly lower index scores in patients with more comorbidities. Effect sizes were mostly small or moderate. Known-groups validity analysis resulted in insignificant difference between groups defined by the majority of localisations both with the 3L and 5L versions. Overall, the 5L was able to better discriminate between known groups of patients based on the number of comorbidities, HS-PGA groups and inguinal localisation (RE > 1), whereas the 3L exhibited a better known-groups validity for body mass index, Hurley stages and gluteal localisation (RE < 1).

**Table 5:** Known-groups validity

	EQ-5D-5L					EQ-5D-3L					RE^*b*^
	*n*	Mean (SD)	Median (Q1-Q3)	*p* value^*a*^	ES	*n*	Mean (SD)	Median (Q1–Q3)	*p* value^*a*^	ES	
Total sample	198	0.76 (0.30)	0.86 (0.71–0.96)	–	–	198	0.78 (0.21)	0.82 (0.75–0.90)	–	–	–
Body mass index (BMI) (*missing n* = *3*)											
Normal or underweight (< 24.9)	38	0.81 (0.20)	0.86 (0.76–0.93)	0.235	0.005	38	0.81 (0.14)	0.81 (0.78–0.90)	0.046	0.022	0.216
Overweight (25.0–29.9)	65	0.78 (0.28)	0.89 (0.71–0.96)			65	0.83 (0.21)	0.88 (0.79–0.98)			
Obese (≥ 30)	92	0.72 (0.33)	0.85 (0.64–0.96)			92	0.75 (0.22)	0.82 (0.60–0.90)			
Comorbidities											
None	106	0.79 (0.27)	0.89 (0.75–0.96)	0.003	0.050	106	0.81 (0.17)	0.85 (0.78–0.90)	0.160	0.032	1.539
1	55	0.80 (0.22)	0.86 (0.76–0.96)			55	0.82 (0.15)	0.82 (0.78–0.90)			
≥ 2	37	0.59 (0.41)	0.74 (0.39–0.88)			37	0.64 (0.31)	0.80 (0.36–0.88)			
Hurley staging (*missing n* = *4*)											
Hurley I	22	0.83 (0.23)	0.89 (0.77–0.97)	0.001	0.068	22	0.83 (0.18)	0.89 (0.78–0.93)	< 0.001	0.071	0.960
Hurley II	79	0.83 (0.21)	0.92 (0.76–0.96)			79	0.83 (0.17)	0.88 (0.80–0.90)			
Hurley III	93	0.67 (0.35)	0.80 (0.57-0.92)			93	0.73 (0.24)	0.80 (0.64–0.88)			
HS-PGA (*missing n* = *7*)											
Clear-minimal	13	0.91 (0.12)	1.00 (0.83–1.00)	< 0.001	0.116	13	0.90 (0.14)	1.00 (0.81–1.00)	< 0.001	0.112	1.036
Mild	37	0.85 (0.17)	0.92 (0.81–0.96)			37	0.84 (0.15)	0.82 (0.80–0.90)			
Moderate	69	0.79 (0.27)	0.88 (0.75–0.96)			69	0.80 (0.19)	0.85 (0.79–0.90)			
Severe	40	0.73 (0.31)	0.81 (0.69–0.92)			39	0.79 (0.16)	0.82 (0.72–0.90)			
Very severe	32	0.53 (0.40)	0.64 (0.20–0.86)			33	0.62 (0.30)	0.72 (0.42–0.81)			
Localisation											
Axillary											
No	44	0.78 (0.25)	0.85 (0.73–0.92)	0.771	0.000	43	0.80 (0.17)	0.80 (0.78–0.90)	0.850	0.000	2.371
Yes	154	0.75 (0.31)	0.87 (0.71–0.96)			155	0.78 (0.22)	0.82 (0.75–0.90)			
Genital											
No	147	0.77 (0.29)	0.88 (0.75–0.96)	0.079	0.016	147	0.79 (0.19)	0.82 (0.75–0.90)	0.491	0.002	6.518
Yes	51	0.71 (0.33)	0.80 (0.60–0.92)			51	0.76 (0.26)	0.82 (0.72–0.90)			
Gluteal											
No	140	0.80 (0.25)	0.88 (0.75–0.96)	< 0.001	0.061	141	0.82 (0.16)	0.85 (0.80–0.90)	< 0.001	0.062	0.986
Yes	58	0.64 (0.36)	0.77 (0.37–0.90)			57	0.68 (0.28)	0.78 (0.54–0.90)			
Inguinal											
No	72	0.85 (0.18)	0.89 (0.79–0.96)	0.004	0.041	72	0.84 (0.14)	0.86 (0.80–0.90)	0.013	0.031	1.314
Yes	126	0.70 (0.34)	0.84 (0.60–0.96)			126	0.75 (0.23)	0.82 (0.64–0.90)			
Perianal											
No	176	0.76 (0.30)	0.87 (0.71–0.96)	0.509	0.002	176	0.79 (0.19)	0.82 (0.78–0.90)	0.140	0.011	0.200
Yes	22	0.73 (0.32)	0.85 (0.70–0.90)			22	0.70 (0.31)	0.79 (0.58–0.88)			
Submammary											
No	174	0.77 (0.28)	0.87 (0.71–0.96)	0.655	0.001	174	0.79 (0.19)	0.82 (0.77–0.90)	0.746	0.001	1.903
Yes	24	0.68 (0.42)	0.82 (0.66–0.96)			24	0.71 (0.33)	0.82 (0.60–0.90)			

*ES* effect size, *HS* hidradenitis suppurativa, *RE* relative efficiency

^a^Mann–Whitney test or Kruskal Wallis test, where a *p* < 0.05 was considered statistically significant

^b^Relative efficiency compared to the EQ-5D-3L

## Discussion

This study aimed to compare the measurement performance of two adult versions (3L and 5L) of the EQ-5D questionnaire in a sample of patients diagnosed with HS. A considerable proportion of HS patients were able to report more problems on the 5L than on the 3L, particularly for mobility, self-care and usual activities dimensions. We found reduced ceiling effects, improved informativity and better known-groups validity for many relevant clinical characteristics for the 5L.

Both acute and chronic pain are common problems reported in HS, and pain medication is usually necessary to improve health outcomes in these patients [69]. Among the five dimensions, the most problems occurred in pain/discomfort, whereby 75.4% (3L) to 77.4% (5L) reported to have ‘any problems’. The pain/discomfort dimension of both the 3L and 5L showed a moderate or moderate-to-strong correlation with current and the worst pain VAS scores suggesting that pain is well captured by the pain/discomfort domain of the EQ-5D. This corroborates with the literature in patients with skin burn, arthritis and Crohn’s disease [60–62].

Ceiling effects were smaller on the 5L for all dimensions with the exception of anxiety/depression, whereby ceiling effects increased by 5.0%. Furthermore, the AD dimension of the 3L showed a stronger correlation with most other outcome measures than that of the 5L. This may be attributable to the different wording used in the descriptor of AD in the Hungarian 3L (3L: ‘anxiety/feeling down’ vs. 5L: ‘anxiety/depression’). This corresponds to previous findings from a 3L-5L comparison study with psoriasis patients in Hungary, whereby the AD dimension of the 3L correlated stronger with both the EQ VAS and DLQI [37].

We found lower mean index scores in the 5L than in the 3L. As the two Hungarian value sets were developed in a parallel valuation study from a common sample using the same preference elicitation technique (i.e. composite time trade-off) and modelling approach, the majority of the differences between index scores are attributable to the differences in wording between the two descriptive systems [47]. This difference between 3L and 5L index scores tended to increase at lower values (Fig. 2). For example, we observed a large difference between the average 3L and 5L index scores in patients with ‘severe’ and ‘very severe’ HS-PGA (0.79 vs. 0.73 and 0.62 vs. 0.53), whereas mean index scores were nearly identical with the two questionnaire versions for the milder severity groups. This suggests that an estimated health gain from an improvement from a ‘very severe’ to ’mild’ HS-PGA health state is substantially larger with the 5L (0.32) than with the 3L (0.22), possibly leading to more favourable cost-effectiveness estimates for HS treatments.

Strengths of the study include the multicentre design, the diverse patient population and the large number of outcome measures available to assess disease severity, pain and HRQoL in HS. The lack of any HS-specific HRQoL instruments available in Hungarian language and the relatively small proportion of patients with lower EQ-5D index scores may be considered as limitations of the study. Moreover, a substantial proportion of patients in the sample had severe HS. On the one hand, we believe that the distribution of the sample across severity groups well represents the treated HS population at large in Hungary, since this was a multicentre study carried out at three academic dermatology clinics. HS patients are almost exclusively treated at these institutions, as systemic and surgical treatments are only available here. On the other hand, the precise epidemiology of HS in Hungary is currently unknown. Compared to the baseline characteristics of HS patients in large international registries [70–73], the proportion of patients with severe HS is higher in our sample that might somewhat limits the external generalizability of the results. A further limitation concerns the positioning of the 3L and 5L within the wider questionnaire caused an ordering effect. The last limitation is that we could not compare the responsiveness and test-retest reliability of the 3L and 5L due to the cross-sectional nature of the study.

In conclusion, our work suggests that the 5L outperforms the 3L version of the EQ-5D in many measurement properties. We recommend the use of the 5L in HS patients across various settings, including clinical care, research and economic evaluations. Future work is recommended to focus on other measurement properties, such as responsiveness, test-retest reliability and comparing the acceptability of the two descriptive systems in terms of ease of understanding and better reflection of health status in this patient population.

## Data Availability

All data of this study are available from the corresponding author upon reasonable request.
